# Definition and Time Evolution of Correlations in Classical Statistical Mechanics

**DOI:** 10.3390/e20120898

**Published:** 2018-11-23

**Authors:** Claude G. Dufour

**Affiliations:** Formerly Department of Physics, Université de Mons-UMONS, Place du Parc 20, B-7000 Mons, Belgium; dufourcg@gmail.com

**Keywords:** information theory, high order correlations, entropy, many-body interactions, irreversibility

## Abstract

The study of dense gases and liquids requires consideration of the interactions between the particles and the correlations created by these interactions. In this article, the *N*-variable distribution function which maximizes the Uncertainty (Shannon’s information entropy) and admits as marginals a set of (*N*−1)-variable distribution functions, is, by definition, free of *N*-order correlations. This way to define correlations is valid for stochastic systems described by discrete variables or continuous variables, for equilibrium or non-equilibrium states and correlations of the different orders can be defined and measured. This allows building the grand-canonical expressions of the uncertainty valid for either a dilute gas system or a dense gas system. At equilibrium, for both kinds of systems, the uncertainty becomes identical to the expression of the thermodynamic entropy. Two interesting by-products are also provided by the method: (i) The Kirkwood superposition approximation (ii) A series of generalized superposition approximations. A theorem on the temporal evolution of the relevant uncertainty for molecular systems governed by two-body forces is proved and a conjecture closely related to this theorem sheds new light on the origin of the irreversibility of molecular systems. In this respect, the irreplaceable role played by the three-body interactions is highlighted.

## 1. Introduction

Shannon’s formula [1,2,3,4] is extensively used in this study. It allows measuring to what extent the outcome of an event described by some probability law is uncertain. This measure is called Uncertainty throughout this work to avoid confusion with entropy. Uncertainty can be defined for equilibrium or nonequilibrium distribution functions and it becomes entropy only at equilibrium (except for a constant factor). If we are given a joint probability distribution *P*(*x*_1_, *x*_2_,…, *x_N_*); the *N* one-variable marginal distributions *P*(*x_i_*) can be obtained by summing the joint distribution over *N* − 1 variables. The Shannon’s formula may be applied to the original joint probability distribution as well as to the product *P*(*x*_1_)*P*(*x*_2_)*P*(*x*_3_)…*P*(*x_N_*). The difference between the two expressions vanishes if all variables are independent and in general, it measures the non-independence among the *N* variables. However, this measure does not indicate to what extent the non-independence among the *N* variables is due to pairs, triples and higher order interactions.

Since the marginal distributions can be derived from *P*(*x*_1_, *x*_2_,…, *x_N_*) and not vice versa, the marginal distributions carry less information than the joint distribution and the uncertainty evaluated from them is higher. Moreover, the lower the order of the marginal distribution, the higher the uncertainty about the system. For example, from knowing the two-variable joint distribution function, to knowing only the one-variable marginal distribution function, the uncertainty of the system increases. From a set of marginal distributions of *P*(*x*_1_, *x*_2_,…, *x_N_*), we may try to build a *N*-particle distribution function *P*’(*x*_1_, *x*_2_,…, *x_N_*). With this sole constraint, many functions *P*’(*x*_1_, *x*_2_,…, *x_N_*) are possible. Following Jaynes [3], in order not to introduce any extra information, the function *P*’ to be chosen is the one that maximizes the uncertainty. In what follows, we call this distribution “uncorrelated”. So, binary correlations exist in *P*(*x*_1_, *x*_2_) if it is different from the distribution function *P*’(*x*_1_, *x*_2_) which is constructed from *P*(*x*_1_) and *P*(*x*_2_), the two marginals of *P*(*x*_1_, *x*_2_). This construction leads to the classical factorization form for the uncorrelated two-variable distribution: *P*’(*x*_1_, *x*_2_) = *P*(*x*_1_*P*(*x*_2_).

In Section 3, when the usual continuous variables of statistical mechanics are used, we verify that the Maxwell-Boltzmann distribution for non-interacting molecules can be expressed exclusively in terms of the single particle distribution function and therefore, that the Maxwell–Boltzmann distribution is uncorrelated, as expected.

In the same way, ternary correlations exist in *P*(*x*_1_, *x*_2_, *x*_3_) if it is not identical to *P*’(*x*_1_, *x*_2_, *x*_3_) which is constructed exclusively from the three 2-variable marginals of *P*(*x*_1_, *x*_2_, *x*_3_). However, the construction of *P*’(*x*_1_, *x*_2_, *x*_3_) is much more involved; the result is no more a product of marginals. For discrete variables, the formal result may be found in References [5,6] and details about the numerical solution can be found in Reference [7].

In Section 4, we show how the method may be applied to binary correlations for the continuous variables of statistical mechanics. Section 4 provides no new result but it is useful as a model to treat ternary correlations. This is done in Section 5 and the equilibrium expression of uncertainty in terms of the first two reduced distribution functions (RDF) is shown to keep its validity out of equilibrium.

The two expressions of the uncertainty for systems without binary correlations or without ternary correlations may be examined from a more physical point of view. The expression of the uncorrelated uncertainty found in Section 4 coincides with the opposite of Boltzmann’s H-function (except for an additive constant term). Since the time of Shannon and Jaynes [1,2,3,4], thermodynamic entropy is generally interpreted as the amount of missing information to define the detailed microscopic state of the system, from the available information, usually the macroscopic variables of classical thermodynamics (e.g., chemical potential *µ*, volume *V* and temperature *T* for a grand canonical ensemble). Out of equilibrium, the opposite of the H-function is the missing information about a system of which we just know the single particle distribution function. Because the equilibrium single particle distribution function carries the same amount of information as the state variables (*V*, *T* and *µ*) the missing information coincides with the thermodynamic entropy at equilibrium (except for a constant factor).

For molecular systems with weak correlations, the amount of missing information to know the microscopic state of the system can be approximated by the uncertainty based on the single distribution function. Section 6 demonstrates that the uncertainty based on the single distribution function of such a system increases while it evolves and correlates. This allows using the uncertainty as an indicator of the irreversibility of weakly coupled dilute systems.

Whenever dense fluids systems (dense gases or liquids systems) are considered, the effects of the interaction potential cannot be neglected. The intermolecular potential is usually determined from the pair correlation function that is closely related to the first two RDFs. Thus, these two RDFs contain the information about the macroscopic parameters of a dense gas. This leads to expect that the best indicator to study the irreversibility of a dense gas system is the missing information about the system of which we just know the first two RDFs, as given by Section 5.

In Section 7.2, the uncertainty based on the first two RDFs is shown to increase while higher order correlations develop, in the same way we do it in Section 6 for the uncertainty based on the first RDF. However, a theorem based on the equations of the Bogoliubov–Born–Green–Kirkwood–Yvon (BBGKY) hierarchy is proved in Section 7.3. It indicates that the time derivative of the uncertainty based on the joint distribution of a molecular system governed by a pairwise additive potential vanishes whenever the system has no three-body correlation. This leads to the conjecture that the two-body uncertainty of a molecular system governed by a pairwise additive potential remains constant. The way to reconcile Section 7.2 and Section 7.3 is considered as one of the concluding elements of Section 8.

Appendix A to Appendix E are intended to make more obvious the relevance of the expression found in Section 5 for the uncertainty of systems without ternary correlation. Two interesting spinoffs of the theory are considered in Appendix D and Appendix E.

## 2. Methods and Notations 

To determine the distribution that maximizes the uncertainty with the constraint(s) about the marginal(s), we use the method of Lagrange multipliers.

When a multivariable joint probability distribution *P*(*x*_1_, *x*_2_,…, *x_N_*) is the sole information about a system, an event, a game or an experiment, its outcomes are unknown. In other words, there is some uncertainty about the outcomes. This uncertainty is measured by Shannon’s formula [1,2]:

(1) $$U=-\underset{x_{1}\ldots \;x_{N}}{\sum }P\left(x_{1},x_{2},\ldots ,x_{N}\right)\;ln\;P\left(x_{1},x_{2},\ldots ,x_{N}\right)$$

The unit of U is the nat (1nat ≈ 1.44 bit) since we choose to use a natural logarithm instead of the usual base 2 logarithm. 

The relevant expression of uncertainty to be used for molecular systems is similar to Equation (1) but it must consider the indistinguishability of the particles and the reduction of uncertainty due to the “uncertainty principle” of quantum mechanics. It is given in (10).

By summing *P*(*x*_1_, *x*_2_,…, *x_N_*) on *n* variables, we obtain the (*N* − *n*)-dimensional marginal distribution functions. 

Particles of molecular systems are characterized by continuous variables (for positions and momenta). Each particle of a molecular system is described by six coordinates: three space-coordinates *q_x_, q_y_, q_z_* for its position and three momentum-coordinates *p_x_, p_y_, p_z_*. For the *i*-th particle, we are going to use the shorthand notations:

(2) $$f\left(i\right)\;\equiv f_{1}\left(q_{i},p_{i}\right)\;\equiv \;f_{1}\left(q_{ix},q_{iy},q_{iz},p_{ix},p_{iy},p_{iz}\right)\;\mathrm{and}\;d\left(i\right)\;=\;dq_{ix}dq_{iy}dq_{iz}dp_{ix}dp_{iy}dp_{iz}$$

Such systems may be described either by their phase-space density (PSD): *P*(1, 2, 3,…, *N*) ≡ *P*($q_{1},p_{1},q_{2},p_{2},q_{3},p_{3}\ldots \ldots .q_{N},p_{N}$) or by means of a set of *s*-body reduced distribution functions (RDF) defined as:

(3) $$f\left(1,2,3,\ldots ,s\right)\;=\underset{N\geq s}{\sum }\frac{N!}{\left(N-s\right)!}\int^{\text{​}}d\left(s+1\right)d\left(s+2\right)\ldots .d\left(N\right)\;P\left(1,\ldots ,N\right),$$

with the normalization condition (for *s* = 0):

(4) $$1=\underset{N\geq 0}{\sum }\int^{\text{​}}d\left(1\right)d\left(2\right)\ldots d\left(N\right)\;P\left(1,\ldots ,N\right)=\;\underset{N\geq 0}{\sum }P_{N}$$

This study assumes that the number of particles *N* is not fixed: all values for *N* are admitted, each with its own probability *P_N_*. In other words, the grand canonical ensemble formalism is used. Additionally, the one-component gas we consider is treated according to the semi-classical approximation. 

The following inversion formulas show that one can switch from the representation in terms of RDFs to the representation in terms of PSDs:

(5) $$P\left(1,2,3,\ldots ,N\right)\;=\frac{1}{N!}\underset{t\geq N}{\sum }\frac{{\left(-1\right)}^{\left(t-N\right)}}{\left(t-N\right)!}\int^{\text{​}}d\left(N+1\right)d\left(N+2\right)\ldots d\left(t\right)\;f\left(1,\ldots ,t\right)$$

Please note that what we call here reduced distribution function (RDF) is often called correlation function and noted “*g*” in literature [8,9,10] while high order RDF can be completely uncorrelated. 

When *f*(1,2) is integrated on moments, the result depends only on the coordinates of the space and only on the relative distance between the particles for a homogeneous isotropic system. This expression divided by the square of the average density:

(6) $$g\;\left(\;\left|q_{1}-q_{2}\right|\;\right)=\;g\left(r\right)\;=\iint^{\text{​}}dp_{1}dp_{2}f\left(1,2\right)\;/{\left(\frac{\langle N\rangle }{V}\right)}^{2}$$

tends to unity when the intermolecular distance *r* increases. It is often called the “pair correlation function” and it is related to the structure factor by a Fourier transform.

## 3. Uncertainty and Correlations for Molecular Systems

### 3.1. Equilibrium Correlations

The way of defining the uncorrelated distribution we have described in the introduction is fully consistent with equilibrium distributions of statistical mechanics: the Maxwell–Boltzmann distribution for non-interacting molecules

(7) $$P_{\left(1,2,3,\ldots ,N\right)}^{eq}\;=C\;e{\;}^{-\;\overset{N}{\underset{i=1}{\sum }}\left(\beta p_{i}^{2}/2m\right)}$$

can easily be expressed exclusively in terms of

(8) $$f_{\left(i\right)}^{eq}=C\prime \;e{\;}^{-\beta p_{i}^{2}/2m}$$

When molecules with a pairwise additive interaction potential are considered, the classical canonical distribution

(9) $$P_{\left(1,2,3,\ldots ,N\right)}^{eq}\;=C\;e{\;}^{-\;\overset{N}{\underset{i=1}{\sum }}\left(\beta p_{i}^{2}/2m\right)\;-\;\overset{N}{\underset{i<j}{\sum }}\left(\beta V_{ij}\right)}$$

can no longer be expressed only in terms of *f^eq^*(1). At equilibrium, potential *V*_12_ is implied in all RDFs, except the first one, in particular in *f^eq^*(1,2). Conversely, *V*_12_ can be expressed in term of *f^eq^*(1,2) [11,12]. Therefore, all PSDs and RDFs can be expressed in terms of $f^{eq}$(1) and $f^{eq}$(1,2). Since only the first two RDFs are involved, we are faced here with binary correlations. 

Similarly, three-body correlations (also called ternary correlations) should appear in the equilibrium distribution of any Hamiltonian system with at least three-body interactions. 

### 3.2. Out of Equilibrium Correlations

For non-equilibrium distributions, we adopt the same basic principle: An uncorrelated probability distribution can be constructed from any probability distribution using exclusively the single-variable distribution function. The uncorrelated probability distribution thus obeys two requirements:

- It maximizes uncertainty;
- The marginal obtained by summing and integrating the distributions over the coordinates of all particles except one is exactly the single-variable distribution.

Similarly, the ternary correlation free distribution can be constructed from the one- and two-variable distribution functions by maximizing the uncertainty with the constraints that the one- and two-variable marginal distribution functions must be conserved.

In this way, for a given *N*-dimensional distribution function, we are able to construct the distribution free of n-order correlation (*n* < *N*) by using their n-order marginal distribution functions. The ratio or the difference between the given function and the constructed distribution free of *n*-order correlation provide a simple measure of the (local) degree of correlation. A more useful global measure of correlation will be defined in Section 4.3. So, due to this precise way of defining correlations, it is possible to distinguish and measure the different levels of correlation within any joint distribution. In a phenomenon described by discrete variables, for example, it becomes possible to verify that ternary correlations can exist in the absence of binary correlations [13].

## 4. Lack of Binary Correlation

### 4.1. The Mathematical Problem

We assume that just the first RDF of some molecular system is known. We attempt to describe the system in terms of the given first RDF. 

Many expressions can be proposed for a pair distribution function f(1,2) compatible with $f_{\left(1\right)}^{given}$, which is the known RDF. Among all these distribution functions, we choose the one that does not include any additional assumptions (i.e., the distribution with the highest possible uncertainty). That distribution is called “free of two-body correlations” or “uncorrelated.” We are therefore led to maximize the relevant uncertainty with constraints. This is typically a Maximum Entropy (MaxEnt) Method introduced by Jaynes [13].

The indistinguishability of the particles and the reduction of uncertainty due to the uncertainty principle of quantum mechanics lead to the semi-classical uncertainty expression we need [8,14,15]:

(10) $$U=-\underset{N\geq 0}{\sum }\int^{\text{​}}d\left(1\right)\ldots d\left(N\right)\;P\left(1,\ldots ,N\right)\;ln\left[N!h^{3N}P\left(1,\ldots ,N\right)\right]$$

As Lagrange multipliers, we need a constant (*λ* + 1) to account for normalization constraint (4) and also a function *λ*(*i*) for the given function $f_{\left(i\right)}^{given}$ to be associated with the constraints (3) taken for *s* = 1. Each Lagrange multiplier *λ*(*i*) depends on the phase-space coordinates of particle *i*. The Lagrange functional is as follows:

(11) $$L\;=-\underset{N\geq 0}{\sum }\int^{\text{​}}d\left(1\right)\ldots d\left(N\right)\;P\left(1,\ldots ,N\right)\ln\left[N!h^{3N}P\left(1,\ldots ,N\right)\right]\;-\left(\lambda +1\right)\left[1-\underset{N\geq 0}{\sum }\int^{\text{​}}d\left(1\right)\ldots d\left(N\right)P\left(1,\ldots ,N\right)\right]\;-\int^{\text{​}}d\left(1\right)\lambda \left(1\right)\left[f_{\left(1\right)}^{given}-\underset{N\geq 1}{\sum }\int^{\text{​}}d\left(2\right)\ldots d\left(N\right)\;N\;P\left(1,\ldots ,N\right)\right]\;=-\underset{N\geq 0}{\sum }\int^{\text{​}}d\left(1\right)\ldots d\left(N\right)\;P\left(1,\ldots ,N\right)\ln\left[N!h^{3N}P\left(1,\ldots ,N\right)\right]\;-\left(\lambda +1\right)\left[1\;-\underset{N\geq 0}{\sum }\int^{\text{​}}d\left(1\right)\ldots d\left(N\right)\;P\left(1,\ldots ,N\right)\right]\;-\int^{\text{​}}d\left(1\right)\lambda \left(i\right)f_{\left(1\right)}^{given}+\underset{N\geq 1}{\sum }\int^{\text{​}}d\left(1\right)\ldots d\left(N\right)\;N\;P\left(1,\ldots ,N\right)\frac{\sum_{i=1}^{N}\lambda \left(i\right)}{N}$$

The symmetrisation operated in the last term ensures that the expression for P(1, …, N) is symmetrical with respect to the coordinates of the *N* particles.

### 4.2. The Solution

A functional derivative of L with respect to P(1,…, N) gives (for any value of *N*):

(12) $$-\;ln\left[N!h^{3N}\;P\left(1,\ldots ,N\right)\right]-1\;+\lambda +1+\sum_{i=1}^{N}\lambda \left(i\right)=0$$

and this leads to the phase-space density P(1,…,N) that can be constructed exclusively from the first RDF:

(13) $$P\left(1,\ldots ,N\right)\;=\;{\tilde{P}}_{\left(1,\ldots ,N\right)}^{\left(1\right)}≝\;\frac{1}{N!\;h^{3N}}e^{\lambda +\overset{N}{\underset{i=1}{\sum }}\lambda (i)}=\;\frac{e^{\lambda }}{N!\;}\overset{N}{\underset{i=1}{\prod }}\frac{e^{\lambda (i)}}{h^{3}}$$

Normalization (4) can be considered to eliminate λ:

(14) $$P\left(1,\ldots ,N\right)={\tilde{P}}_{\left(1,\ldots ,N\right)}^{\left(1\right)}=\frac{\frac{1}{N!\;}\prod_{i=1}^{N}\frac{e^{\lambda (i)}}{h^{3}}}{\sum_{n=0}^{\infty }\frac{1}{n!\;}\int^{\text{​}}d\left(1\right)\ldots d\left(n\right)\prod_{i=1}^{n}\frac{e^{\lambda (i)}}{h^{3}}}\;=\;\frac{\frac{1}{N!\;}\prod_{i=1}^{N}\frac{e^{\lambda (i)}}{h^{3}}}{\sum_{n\geq 0}^{}\frac{1}{n!\;}{\left[\int^{\text{​}}d\left(i\right)\frac{e^{\lambda (i)}}{h^{3}}\right]}^{n}}$$

The constraint (3) for *s* = 1 becomes:

(15) $$f\left(1\right)=\underset{N\geq 1}{\sum }N\;\frac{\int^{\text{​}}d\left(2\right)\ldots d\left(N\right)\frac{1}{N!\;}\prod_{i=1}^{N}\frac{e^{\lambda (i)}}{h^{3}}}{\sum_{n\geq 0}^{}\frac{1}{n!\;}{\left[\int^{\text{​}}d\left(i\right)\frac{e^{\lambda (i)}}{h^{3}}\right]}^{n}}=\frac{e^{\lambda (1)}}{h^{3}}\frac{\sum_{N-1\geq 0}^{}\frac{\;1\;}{\left(N-1\right)!}{\left[\int^{\text{​}}d\left(i\right)\frac{e^{\lambda (i)}}{h^{3}}\right]}^{N-1}}{\sum_{n\geq 0}^{}\frac{1}{n!\;}{\left[\int^{\text{​}}d\left(i\right)\frac{e^{\lambda (i)}}{h^{3}}\right]}^{n}}\;=\frac{e^{\lambda (1)}}{h^{3}}$$

After eliminating *λ*(*i*) between Equations (14) and (15), the usual factored form of phase-space density is recovered [4,15]:

(16) $${\tilde{P}}_{\left(1,\ldots ,N\right)}^{\left(1\right)}\;=\;\frac{\frac{\;1}{N!}\prod_{i=1}^{N}\;f\left(i\right)}{\sum_{N\geq 0}^{}\int^{\text{​}}\frac{d\left(1\right)\ldots d\left(N\right)}{N!}\prod_{i=1}^{N}f\left(i\right)}\;=\;\frac{\frac{\;1}{N!}\prod_{i=1}^{N}\;f\left(i\right)}{\sum_{N\geq 0}^{}\frac{<N{>}^{N}}{N!}}\;=\frac{e^{-\langle N\rangle }}{N!}\overset{N}{\underset{i=1}{\prod }}f\left(i\right)$$

where <*N*> is defined as the mean number of particles:

(17) $$\langle N\rangle \;=\int^{\text{​}}d\left(1\right)\;f\left(1\right)$$

The same result is obtained when maximizing the expression of Gibbs-Shannon uncertainty (10) in terms of RDFs as can be found in [8,9,16]. 

### 4.3. Some Consequences of the Solution

Equations (3) and (16) yield the usual factorization formula:

(18) $${\tilde{f}}_{\left(1,2,3,\ldots ,s\right)}^{\left(1\right)}\;=\;f\left(1\right)f\left(2\right)\;f\left(3\right)\ldots \;f\left(s\right)$$

So, factorization is a consequence of the basic principle stating that a *s*-body RDF is uncorrelated if it does not contain more information than that included in the first RDF.

Extra normalization constants appear in this factorization formula when the formalism of the canonical ensemble is used. For example, because of the normalization of *f*(1) and *f*(1,2), respectively to $N_{c}\;\mathrm{and}\;N_{c}\left(N_{c}-1\right)$, Equation (18) becomes:

(19) $${\tilde{f}}_{can\;\left(1,2\right)}^{\left(1\right)}\;=\;\frac{N_{c}\left(N_{c}-1\right)}{N_{c}{}^{2}}\;f\left(1\right)f\left(2\right)$$

However, the extra factor disappears when the thermodynamic limit is applied (*Nc* → ∞, *V* → ∞, *Nc/V* = Constant).

Substituting *P*(1,…, *N*) in Equations (10) with (16) yields the uncertainty of the system when it is described by the first RDF. This means that nothing is known about its correlations or that the system is free of any correlations [14,15].

(20) $$U_{1}\;\equiv \;U\left[f\left(1\right)\right]\;=\;-\;\int^{\text{​}}d\left(1\right)\left[f\left(1\right)lnh^{3}f\left(1\right)-\;f\left(1\right)\right]$$

The way in which ${\tilde{P}}_{\left(1,\ldots ,N\right)}^{\left(1\right)}$ has been defined implies that uncertainty *U*_1_ must be greater than *U*, as given by Equation (10):

(10) $$U_{1}\;\geq \;U$$

This fundamental inequality can be proved from the basic inequality *ln x* ≤ *x* − 1, replacing *x* by ${\tilde{P}}_{\left(1,\ldots ,N\right)}^{\left(1\right)}/P_{\left(1,\ldots ,N\right)}$, multiplying both sides by $P_{\left(1,\ldots ,N\right)}$, integrating over all variables and finally, summing over *N*.

So, to find out if a distribution function *P*_(1,..., *N*)_ is correlated, just calculate the first RDFs and use then to compute ${\tilde{P}}_{\left(1,\ldots ,N\right)}^{\left(1\right)}$. If $P_{\left(1,\ldots ,N\right)}$ ≡ ${\tilde{P}}_{\left(1,\ldots ,N\right)}^{\left(1\right)}$, the $P_{\left(1,\ldots ,N\right)}$ is uncorrelated; all RDFs are then factored and *U*_1_ = *U*. At the contrary, *U*_1_ − *U* > 0 means that function $P_{\left(1,\ldots ,N\right)}$ is correlated and we can define *U*_1_ − *U* as the measure of the correlations (from all orders).

### 4.4. The Special Case of Equilibrium State

When the equilibrium distribution

(22) $$f\left(1\right)\;=\;\frac{\langle N\rangle }{V}{\left(\frac{1}{2\pi \;m\;k_{B}T}\right)}^{3/2}e^{-\;\frac{p_{x}{}^{2}+p_{y}{}^{2}+p_{z}{}^{2}}{2m\;k_{B}T}}$$

is inserted inside (20), the Sackur–Tetrode formula is recovered [14,17]:

(23) $$S=k_{B}U^{eq}=\langle N\rangle k_{B}\left\{\;ln\left[\frac{V}{\langle N\rangle }{\left(\frac{2\pi \;m\;k_{B}T}{h^{2}}\right)}^{3/2}\right]+\frac{5}{2}\;\right\}\;\;=\;\langle N\rangle k_{B}\left\{\;ln\left[\frac{V}{\langle N\rangle }{\left(\frac{4\pi \;m\;E}{3\langle N\rangle h^{2}}\right)}^{3/2}\right]+\;\frac{5}{2}\right\}$$

At equilibrium, uncertainty identifies with entropy (with the exception of the multiplicative Boltzmann’s constant) and the following thermodynamic equations may be checked explicitly for a monatomic ideal gas:

(24) $${\left(\frac{\partial S}{\partial V}\right)}_{E,\langle N\rangle }\;=\;\frac{\langle N\rangle k_{B}}{V}\;=\;\frac{\;p\;}{T}$$

(25) $${\left(\frac{\partial S}{\partial E}\right)}_{V,\langle N\rangle }\;=\;\frac{3\langle N\rangle k_{B}}{2E}=\;\frac{1}{T}$$

Out of equilibrium, there is no reason that these equations hold. This is why the term “entropy” should be avoided for non-equilibrium states.

The Sackur–Tetrode formula can no more be used when moderately-dense gas systems are considered because correlations are no more negligible in this case. A description in terms of both *f*(1) and *f*(1,2) is necessary since, the lowest order RDF that can be correlated is *f*(1,2).

We will now construct the distribution functions that contain two-body correlations but no three-body correlations.

## 5. Lack of Ternary Correlation

### 5.1. The Mathematical Problem

The known RDFs are called $f_{\left(1\right)}^{given}$ and $f_{\left(1,2\right)}^{given}$ and the searched phase-space density is ${\tilde{P}}_{\left\{N\right\}}^{\left(2\right)}$, based on the knowledge of the first two RDFs instead of just the first one. Its derivation follows exactly the lines used for ${\tilde{P}}_{\left\{N\right\}}^{\left(1\right)}$.

${\tilde{P}}_{\left\{N\right\}}^{\left(2\right)}$ must maximize the Gibbs-Shannon uncertainty (10) with three constraints expressed by Equation (3) as *s* = 0, 1 and 2. We need an additional Lagrange multiplier function like ½*λ*(*i*,*j*) which depends on the phase-space coordinates of two particles. Again, a symmetrisation is carried out as one looks for a symmetrical solution with respect to all particles:

(26) $$\begin{array}{cc} L= & -\underset{N\geq 0}{\sum }\int^{\text{​}}d\left(1\right)\ldots d\left(N\right)\;P\left(1,\ldots ,N\right)ln\left[N!h^{3N}P\left(1,\ldots ,N\right)\right]\\ & -\;\left(\lambda +1\right)\left[1-\;\underset{N\geq 0}{\sum }\int^{\text{​}}d\left(1\right)\ldots d\left(N\right)\;P\left(1,\ldots ,N\right)\right]\\ & -\int^{\text{​}}d\left(1\right)\;\lambda \left(1\right)\left[f_{\left(1\right)}^{given}-\underset{N\geq 1}{\sum }\int^{\text{​}}d\left(2\right)\ldots d\left(N\right)\;\;\;N\;\;\;P\left(1,\ldots ,N\right)\right]\\ & -\int^{\text{​}}d\left(1\right)d\left(2\right)\frac{\lambda \left(1,2\right)}{2}\left[f_{\left(1,2\right)}^{given}-\underset{N\geq 2}{\sum }\int^{\text{​}}d\left(3\right)\ldots d\left(N\right)\;\;\;\frac{N!}{\left(N-2\right)!}\;\;P\left(1,\ldots ,N\right)\right] \end{array}$$

(27) $$\begin{array}{ll} = & -\underset{N\geq 0}{\sum }\int^{\text{​}}d\left(1\right)\ldots d\left(N\right)P\left(1,\ldots N\right)ln\left[N!h^{3N}P\left(1,\ldots ,N\right)\right]\\ & -\left(\lambda +1\right)\left[1-\;\underset{N\geq 0}{\sum }\int^{\text{​}}d\left(1\right)\ldots d\left(N\right)\;P\left(1,\ldots ,N\right)\right]\\ & -\int^{\text{​}}d\left(1\right)\;\lambda \left(1\right)f_{\left(1\right)}^{given}\;+\underset{N\geq 1}{\sum }\int^{\text{​}}d\left(1\right)\ldots d\left(N\right)\;P\left(1,\ldots ,N\right)\overset{N}{\underset{i=1}{\sum }}\lambda \left(i\right)\\ & -\int^{\text{​}}d\left(1\right)d\left(2\right)\frac{\lambda \left(1,2\right)}{2}f_{\left(1,2\right)}^{given}\;+\underset{N\geq 2}{\sum }\int^{\text{​}}d\left(1\right)\ldots d\left(N\right)\;P\left(1,\ldots ,N\right)\overset{N-1}{\underset{i=1}{\sum }}\overset{N}{\underset{j=i+1}{\sum }}\lambda \left(i,j\right) \end{array}$$

After functional differentiation, we get the following factored form of P(1,…, N):

(28) $$P\left(1,\ldots ,N\right)={\tilde{P}}_{\left(1,\ldots ,N\right)}^{\left(2\right)}\equiv \;\frac{\;1}{N!\;h^{3N}}e^{\lambda +\overset{N}{\underset{i=1}{\sum }}\lambda (i)\;+\;\overset{N-1}{\underset{i=1}{\sum }}\overset{N}{\underset{j=i+1}{\sum }}\lambda (i,j)}$$

### 5.2. Some Consequences of the Solution

The *s*-particle RDF when constructed from the information included in the first two RDFs will be denoted by ${\tilde{f}}_{\left(1,2,\ldots ,s\right)}^{\left(2\right)}$ will denote:

(29) $${\tilde{f}}_{\left(1,2,3,\ldots ,s\right)}^{\left(2\right)}\;=\;\underset{N\geq s}{\sum }\frac{N!}{\left(N-s\right)!}\int^{\text{​}}d\left(s+1\right)\ldots d\left(N\right)\;{\tilde{P}}_{\left(1,\ldots ,N\right)}^{\left(2\right)}.$$

The uncertainty about a system known through its first two RDFs is:

(30) $$U_{2}\;\equiv \;U\left[{\tilde{P}}_{\left\{N\right\}}^{\left(2\right)}\right]=-\underset{N\geq 0}{\sum }\int^{\text{​}}d\left(1\right)\ldots d\left(N\right)\;{\tilde{P}}_{\left(1,\ldots ,N\right)}^{\left(2\right)}ln\left[N!h^{3N}\;{\tilde{P}}_{\left(1,\ldots ,N\right)}^{\left(2\right)}\right].$$

Due to the way ${\tilde{P}}_{\left(1,\ldots ,N\right)}^{\left(2\right)}$ is defined, *U*_2_ must be greater than the general uncertainty when all RDFs and all PSDs *P*(1,..., *N*) are known:

(31) $$U_{2}\;=\;U\left[{\tilde{P}}_{\left(1,\ldots ,N\right)}^{\left(2\right)}\right]\;\geq \;U\left[P_{\left(1,\ldots ,N\right)}\right]\;=\;U.$$

The factored form (28) of ${\tilde{P}}_{\left\{N\right\}}^{\left(2\right)}$ makes it easy to prove this inequality by the method that led to Equation (21). Moreover Equation (31) becomes an equality only if $P_{\left(1,\ldots ,N\right)}\;=\;{\tilde{P}}_{\left(1,\ldots ,N\right)}^{\left(2\right)}$.

Here again, *U*_2_ − *U* measures the correlations of order higher than two. Since *U*_1_ − *U* measures correlations of order two, three, …, the difference of the two quantities (*U*_1_ − *U*) − (*U*_2_ − *U*) = *U*_1_ − *U*_2_ measures exclusively the binary correlations and it can be shown that this measure is also non-negative.

As long as the Lagrange multipliers are undetermined, Equations (28), (29) and (30) remain formal. Only the first Lagrange multiplier *λ* is easily determined (due to the normalization condition). The first terms of the series expansion of *λ*(*i*) and *λ*(*i*,*j*) can be obtained by functionally derivating the Lagrangian with respect to the RDFs (See Appendix C). However, this method is rather tedious and we prefer to use a diagrammatic method which achieves the global result.

### 5.3. A More Explicit Solution - Uncertainty in Terms of f(1) and f(1,2)

Given the normalization condition, Equation (28) can be written:

(32) $${\tilde{P}}_{\left\{N\right\}}^{\left(2\right)}\equiv \;{\tilde{P}}_{\left(1,\ldots ,N\right)}^{\left(2\right)}\;=\;\frac{\frac{1}{N!\;h^{3N}}\;e{\;}^{\sum_{i=1}^{N}\lambda (i)\;+\;\sum_{i=1}^{N-1}\sum_{j=i+1}^{N}\lambda (i,j)}}{\sum_{N\geq 0}^{}\int^{\text{​}}\frac{\;d\left(1\right)\ldots d\left(N\right)}{N!\;h^{3N}}e{\;}^{\sum_{i=1}^{N}\lambda (i)\;+\;\sum_{i=1}^{N-1}\sum_{j=i+1}^{N}\lambda (i,j)}}.$$

The Lagrange multipliers λ(i) and λ(i,j) can be found by eliminating ${\tilde{P}}_{\left(1,\ldots ,N\right)}^{\left(2\right)}$ from Equation (32) and the two constraints equations:

(33) $$f\left(1\right)\;=\underset{N\geq 1}{\sum }\frac{N!}{\left(N-1\right)!}\int^{\text{​}}d\left(2\right)d\left(3\right)\ldots d\left(N\right){\tilde{P}}_{\left(1,\ldots ,N\right)}^{\left(2\right)}$$

(34) $$f\left(1,2\right)\;=\underset{N\geq 2}{\sum }\frac{N!}{\left(N-2\right)!}\int^{\text{​}}d\left(3\right)d\left(4\right)\ldots d\left(N\right){\tilde{P}}_{\left(1,\ldots ,N\right)}^{\left(2\right)}.$$

This elimination work has been conducted by various authors [11,18,19]. In fact, these authors were interested only in the study of equilibrium systems and their starting equations correspond to ours by the substitution:

(35) $$\lambda \left(i,j\right)\;\leftarrow \;-\beta \;V_{ij}$$

(36) $$\mathrm{and}\;\lambda (i)\;\leftarrow \;-\beta \;\left[-µ\;+\frac{p_{i}^{2}}{2m}\;+\;V_{i}\right],$$

where $\beta =1/(k_{B}T)$, *µ* is the chemical potential and *V_i_* ≡ *V*(***q_i_***) stands for an external potential acting on a particle located at ***q_i_***. From a mathematical point of view, there are two significant differences:

a) Function λ(i,j) depends on space and momentum coordinates (***q**_i_*, ***q**_j_*, ***p**_i_*, ***p**_j_*) and not only on space coordinates (***q**_i_*, ***q**_j_*).
b) *d*(*i*) in Equations (33) and (34) means *d**q**_i_d**p**_j_* and not just *d**q**_i_*.

Since the diagrammatic analysis in References [18] and [19] does not depend on the number of variables and the real variables inside, *λ*(*i*,*j*) and *λ*(*i*), we claim that their derivation is not invalidated by changes (a) and (b) so that their results are still valid. The uncertainty on the system known by its first two RDFs is [11,18]:

(37) $$\begin{array}{cc} U_{2}= & -\int^{\text{​}}d\left(1\right)\left[f\left(1\right)\ln h^{3}f\left(1\right)-f\left(1\right)\right]\\ & -\frac{1}{2}\int^{\text{​}}d\left(1\right)d\left(2\right)\left[f\left(1,2\right)\left(\ln\frac{f\left(1,2\right)}{f\left(1\right)f\left(2\right)}-1\right)+f\left(1\right)f\left(2\right)\right]\\ & +\frac{1}{6}\int^{\text{​}}d\left(1\right)d\left(2\right)d\left(3\right)f\left(1\right)f\left(2\right)f\left(3\right)\left[C_{12}C_{13}C_{23}\right]\\ & +\frac{1}{24}\int^{\text{​}}d\left(1\right)d\left(2\right)d\left(3\right)d\left(4\right)f\left(1\right)f\left(2\right)f\left(3\right)f\left(4\right)\left[-3C_{13}C_{14}C_{23}C_{24}+C_{12}C_{13}C_{14}C_{23}C_{24}C_{34}\right]\\ & -\ldots , \end{array}$$

where the factor *C_ij_* is defined by

(38) $$C_{ij}\;≝\;\left(\frac{f\left(i,j\right)}{f\left(i\right)f\left(j\right)}-1\right).$$

Diagrams are usually used to shorten the writings:(39)
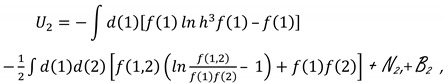

where 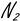 = sum of polygonal (also called nodal) diagrams with alternate signs
(40)
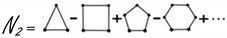

and 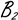 = sum of all more than doubly connected diagrams, that is, connected diagrams in which there exist at least three independent paths to reach any circle or dot in the diagram from any other circles. These paths are independent of each other if they do not involve a common intermediate circle.

(41)
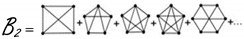


Diagrams composed of links and white or black circles should be calculated according to the following rules:

- To each heavy black circle, assign a label j that is different from all other labels that are already present and associate a factor *f*(*j*) with that circle.
- With each line *i*-*j*, associate a factor *C_ij_* defined by (38).
- The arguments (*i*) of each heavy black circle *i* should be integrated and the result should be divided by the number of symmetries in the diagram.

The expressions of *λ*(1) and *λ*(1,2) in terms of *f*(1) and *f*(1,2) are given in Equations (A9) and (A12).

We verify in Appendix A that *U*_2_, as given by Equation (39) is consistent with the Gibbs entropy of a plasma out of equilibrium. We also show, in Appendix D and Appendix E the advantages of this method in deriving the Kirkwood superposition approximation (KSA) [20] and the Fisher-Kopeliovich closure [21] (see also references [10,22]).

The most important point of this Section is to realize that when only the first two RDFs of a system are known, the expression of uncertainty exists and is well-defined, even though it is complicated.

## 6. Time Evolution of Correlations in a Weakly Correlated Gas System

A system is weakly correlated and Uncertainty *U*_1_ is the relevant indicator for studying its irreversibility, only if the potential energy due to intermolecular forces is small compared to the kinetic energy of the particles. This is the case if the depth of the potential well is low compared to the average energy of a particle (high temperature assumption). In addition, the density should be small enough that the average distance between the particles is large and they stay out of the range of the interaction potential most of the time (low density assumption).

We now show that uncertainty *U*_1_ increases whenever correlations are created.

A Hamiltonian system can be considered at two different times: *t*_0_ and *t*. According to Liouville’s theorem, the uncertainty, *U*, is constant between the instant t_0_ and the instant *t*:

(43) *U*(*t*) = *U*(*t*_0_)

If the system is free of correlations at the initial time, its uncertainty, *U*(*t*_0_) reduces to *U*_1_[*f*(1,*t*_0_)] given by (20):

(43) $$U\left[f\left(1,t_{0}\right)\right]\;\equiv \;U_{1}\left(t_{0}\right)\;=-\;\int^{\text{​}}d\left(1\right)\left[f\left(1,t_{0}\right)lnf\left(1,t_{0}\right)-\;f\left(1,t_{0}\right)\right].$$

By assuming that, at time *t*, correlations have been created, Equation (21) states that

(44) $$U_{1}\left(t\right)\equiv \;U\;\left[f\left(1,t\right)\right]>U\left(t\right),$$

and by collecting (42), the left part of (43) and (44) clearly shows that uncertainty U [f(1,t)] increases from time *t*_0_ to time *t* if correlations (of any order) are created during the same period of time:

(45) $$U\left[f\left(1,t\right)\right]>U\left(t\right)\;=\;U\left(t_{0}\right)\;=\;U\left[f\left(1,t_{0}\right)\right].$$

If no correlation is created, inequality (45) becomes equality; *U*_1_ remains constant.

## 7. Evolution of Correlations in Dense Gas Systems

### 7.1. Relevant Expression of Uncertainty for Dense Gas Systems

From the first RDF, we can draw information about density, volume and kinetic energy of a gas. On the other hand, in dense gas systems, we do know that besides the translational energy of the molecules, a mean potential energy term appears and the perfect gas pressure formula must be corrected to take into account the intermolecular attractions [4]. If we remember that the intermolecular potential is determined from the pair correlation function (itself determined from neutron or X-ray scattering experiments), we will realize that the first two RDFs $f_{\left(1\right)}^{eq}\;\mathrm{and}\;f_{\left(1,2\right)}^{eq}$ bear the information about the relevant macroscopic parameters of a dense gas system. Consistently, the amount of information that is still missing to define the detailed microscopic state of the system is no more *U*_1_ but *U*_2_ (the uncertainty about the system, the first two RDFs *f*(1) and *f*(2) being known).

### 7.2. Uncertainty U_2_ Increases Whenever Correlations of Order Higher Than Two Are Created

From Equation (31) and arguments similar to those used in Section 6, we can draw similar conclusions:

(46) $$U_{2}\left[\;f\left(1,t\right),f\left(2,t\right),f\left(1,2,t\right)\right]\;\geq \;U\left(t\right)\;=\;U\left(t_{0}\right)\;=\;U_{2}\left[f\left(1,t_{0}\right),f\left(2,t_{0}\right),f\left(1,2,t_{0}\right)\right].$$

Let us examine the meaning of this equation for some system which evolves from time *t*_0_ to time *t*. According to Equation (46), initial and final values of *U*_2_ may coincide. This happens, for instance if the system is already in an equilibrium state at time *t*_0_. For a non-stationary transformation of the system, the value of *U*_2_ at time *t* may differ from the value of *U*, at the same time. Due to its particular structure, *U*_2_ can only be greater or equal to *U* as was indicated in Equation (31). 

Usually, changes in the distribution functions of an evolving system are called correlations and it is said that *U*_2_ increases as correlations settle, without being able to indicate the type of the correlations that are created. With the information-theoretic definition of correlations developed in Section 5, the type of the correlations can be specified: the difference *U*_2_ − *U* measures the correlations of order higher than two. So, whenever correlations of order higher than two are created, uncertainty *U*_2_ increases.

As a consequence, for systems of molecules with pairwise interactions, *U*_2_ meets the two requirements that allow it to be used as an indicator of irreversibility:

- *U*_2_ becomes the equilibrium entropy for the equilibrium distributions.
- Starting from a system which is free of correlations of order higher than two, uncertainty *U*_2_ increases if correlations of order higher than two are created in the same time.

However, the Gibbs entropy constancy property, usually expressed in a *N*-particle description could also appear in a two-particle description, as shown in the next Section and this can preclude the development of correlations of order higher than two and upset our strategy for describing irreversibility.

### 7.3. Time Dependence of U_2_ from the BBGKY Hierarchy

Under the same assumptions of pairwise interactions and no initial correlation of order greater than two, we can prove that the time derivative of *U*_2_ vanishes at the initial time and this property could remain valid for all subsequent times.

**Hypotheses:**  *At some time t_0_, there is no three-body correlation:*

(47) $$P\left(1,\ldots ,N\right){|}_{t_{0}}\equiv P_{\left\{N\right\}}{|}_{t_{0}}={\tilde{P}}_{\left\{N\right\}}^{\left(2\right)}{|}_{t_{0}}\;\mathrm{or}\;f\left(1,2,3\right){|}_{t_{0}}=\;{\tilde{f}}_{\left(1,2,3\right)}^{\left(2\right)}{|}_{t_{0}}=\overset{}{\underset{N}{\sum }}\frac{N!}{\left(N-3\right)!}\int^{\text{​}}d\left(4\right)d\left(5\right)\ldots \;d\left(N\right){\tilde{P}}_{\left\{N\right\}}^{\left(2\right)}{|}_{t_{0}}.$$

*For two-body interactions, the Liouvillian is:*

(48) $$L=L{}^{\circ}+\;L^{\prime }\equiv \left(\overset{N}{\underset{i=1}{\sum }}L_{i}^{{}^{\circ}}+\overset{N-1}{\underset{j=1}{\sum }}\overset{N}{\underset{k=j+1}{\sum }}L_{jk}^{\prime }\right)$$

*and the phase-space densities (32) to be used:*

(49) $$ln\;{\tilde{P}}_{\left\{N\right\}}^{\left(2\right)}=\;\lambda \prime +\sum_{i=1}^{N}\lambda (i)+\sum_{i=1}^{N-1}\sum_{j=i+1}^{N}\lambda (i,j);\;\lambda \prime =\lambda +ln\frac{1}{N!h^{3N}},\ldots ,$$

*with three constraints defining the λs:*

(50) $$\sum_{N}\int^{\text{​}}d\left(1\right)d\left(2\right)\ldots d\left(N\right){\tilde{P}}_{\left\{N\right\}}^{\left(2\right)}\;=1,$$

(51) $$\sum_{N}\;N\int^{\text{​}}d\left(2\right)d\left(3\right)\ldots d\left(N\right){\tilde{P}}_{\left\{N\right\}}^{\left(2\right)}\;=f\left(1\right),$$

(52) $$\sum_{N}\;N\left(N-1\right)\int^{\text{​}}d\left(3\right)d\left(4\right)\ldots d\left(N\right){\tilde{P}}_{\left\{N\right\}}^{\left(2\right)}\;=f\left(1,2\right),$$

*and boundary conditions:*

(53) $${\tilde{P}}_{\left\{N\right\}}^{\left(2\right)}\;=\;0\;\mathrm{for}\;p\;\mathrm{or}\;q\;=\;\pm \infty .$$

**Thesis:**  (54) $$\partial_{t}U_{2}{|}_{t_{0}}\equiv \;\partial_{t}U\left[{\tilde{P}}_{\left\{N\right\}}^{\left(2\right)}\right]{|}_{t_{0}}\equiv \left[-\;\partial_{t}\underset{N}{\sum }\int^{\text{​}}d\left(1\right)d\left(2\right)\ldots d\left(N\right){\tilde{P}}_{\left\{N\right\}}^{\left(2\right)}ln\;N!h^{3N}{\tilde{P}}_{\left\{N\right\}}^{\left(2\right)}\right]{|}_{t_{0}}\;=0.$$

**Proof.**  Time-differentiating Equation (30), and taking into account Equations (49) and (50), yield:

(55) $$\partial_{t}U_{2}=-\sum_{N}\int^{\text{​}}d\left(1\right)d\left(2\right)\ldots d\left(N\right)\left[1+\lambda \prime +ln\;N!h^{3N}+\sum_{i}^{N}\lambda (i)+\sum_{i=1}^{N-1}\sum_{j=i+1}^{N}\lambda (i,j)\right]\partial_{t}{\tilde{P}}_{\left\{N\right\}}^{\left(2\right)}.$$

All λ(i) terms yield the same contribution to the integral and ∑λ(i) may be replaced by for example, λ(1) multiplied by N. Similarly, the terms ∑∑λ(i,j) will be replaced by λ(1,2) N(N − 1)/2:

(56) $$\begin{array}{cc} \partial_{t}U_{2}= & -\;\left(1+\;\lambda \prime +\;ln\;N!h^{3N}\right)\partial_{t}\underset{N}{\sum }\int^{\text{​}}d\left(1\right)d\left(2\right)\ldots d\left(N\right){\tilde{P}}_{\left\{N\right\}}^{\left(2\right)}\\ & -\int^{\text{​}}d\left(1\right)\lambda (1)\partial_{t}\underset{N}{\sum }N\int^{\text{​}}d\left(2\right)d\left(3\right)\ldots d\left(N\right){\tilde{P}}_{\left\{N\right\}}^{\left(2\right)}\\ & -\;½\int^{\text{​}}d\left(1\right)d\left(2\right)\lambda (1,2)\partial_{t}\sum_{N}N\left(N-1\right)\int^{\text{​}}d\left(3\right)\ldots d\left(N\right){\tilde{P}}_{\left\{N\right\}}^{\left(2\right)}. \end{array}$$

Due to normalization (50), the first term disappears. In the other terms, we recognize the definitions (51) & (52) of f(1) & f(1,2):

(57) $$\partial_{t}U_{2}\;=-\;\int d\left(1\right)\lambda (1)\partial_{t}\;f\left(1\right)\;-\;½\;\int d\left(1\right)d\left(2\right)\lambda (1,2)\partial_{t}\;f\left(1,2\right).$$

This intermediate result could also be achieved by the fact that(1) and ½λ(1,2) are the functional derivatives of U_2_ with respect to f(1) and f(1,2).

As shown by Equation (3), these two RDFs are obtained by integrating the PSD over the variables of (N−1) and (N−2) particles, respectively. The equations giving the expression of their time derivative (the so-called hierarchy BBGKY) [14], are obtained by integrating the Liouville equation over the same variables:

(58) $$\partial_{t}f\left(1\right)=\;L_{1}^{{}^{\circ}}f\left(1\right)\;+\;\int d\left(1\right)d\left(2\right)\;L_{12}^{\prime }f\left(1,2\right),$$

(59) $$\partial_{t}f\left(1,2\right)=(L_{12}^{{}^{\circ}}+L_{12}^{\prime })f\left(1,2\right)+\int d\left(3\right)\left(L_{13}^{\prime }+L_{23}^{\prime }\right)f\left(1,2,3\right).$$

By substitution, one achieves:

(60) $$\begin{array}{cc} \partial_{t}U_{2}= & -\int d\left(1\right)\left(1\right)\left[L_{1}^{{}^{\circ}}f\left(1\right)+\;d\left(1\right)d\left(2\right)L_{12}^{\prime }f\left(1,2\right)\right]\\ & -½\;\int d\left(1\right)d\left(2\right)\left(1,2\right)\left[(L_{12}^{{}^{\circ}}+L_{12}^{\prime })f\left(1,2\right)+\int d\left(3\right)\left(L_{13}^{\prime }+L_{23}^{\prime }\right)f\left(1,2,3\right)\right], \end{array}$$

and by integrating by parts:

(61) $$\begin{array}{cc} \partial_{t}U_{2}= & \int d\left(1\right)\;\left[\;f\left(1\right)L_{1}^{{}^{\circ}}\lambda (1)\;+\;\int d\left(1\right)d\left(2\right)\;f\left(1,2\right)L_{12}^{\prime }\lambda (1)\right]\;\\ & +\;½\;\int d\left(1\right)d\left(2\right)\left[f\left(1,2\right)(L_{12}^{{}^{\circ}}+L_{12}^{\prime })\lambda (1,2)+\int d\left(3\right)f\left(1,2,3\right)\left(L_{13}^{\prime }+L_{23}^{\prime }\right)\lambda (1,2)\right] \end{array}$$

Now the time derivatives have been ruled out, RDFs f(1) and f(1,2) may be eliminated in favour of${\tilde{P}}_{\left\{N\right\}}^{\left(2\right)}$ by using Equations (51) and (52) in the opposite direction. If we consider the equation at time t_0_, the non-correlation assumption (47) allows substitutingf(1,2,3) with ${\tilde{f}}_{\left(1,2,3\right)}^{\left(2\right)}$ to finally reintroduce${\tilde{P}}_{\left\{N\right\}}^{\left(2\right)}$:

(62) $$\begin{array}{cc} \partial_{t}U_{2}{|}_{t_{0}}= & \underset{N}{\sum }\int^{\text{​}}d\left(1\right)\ldots d\left(N\right){\tilde{P}}_{\left\{N\right\}}^{\left(2\right)}\left[NL_{1\;}^{{}^{\circ}}\lambda (1)+N\left(N-1\right)\;L_{12\;}^{\prime }\lambda (1)+\frac{N\left(N-1\right)}{2}\;\left(L_{1}^{{}^{\circ}}+L_{2}^{{}^{\circ}}+L_{12}^{\prime }\right)\;\lambda (1,2)\right]\\ & +\underset{N}{\sum }\int^{\text{​}}d\left(1\right)\ldots d\left(N\right){\tilde{P}}_{\left\{N\right\}\;}^{\left(2\right)}\left[L_{13}^{\prime }\lambda (1,2)+\;L_{23}^{\prime }\lambda (1,2)\right]\frac{N\left(N-1\right)\left(N-2\right)}{2} \end{array}$$

The numerical factors are the ones needed to present the result as a product of multiple summations:

(63) $$\partial_{t}U_{2}{|}_{t_{0}}=\underset{N}{\sum }\int^{\text{​}}d\left(1\right)\ldots d\left(N\right){\tilde{P}}_{\left\{N\right\}}^{\left(2\right)}\left[\overset{N}{\underset{j=1}{\sum }}L_{j}^{{}^{\circ}}+\overset{N-1}{\underset{j=1}{\sum }}\overset{N}{\underset{k=j+1}{\sum }}L_{jk}^{\prime }\right]\left[\overset{N}{\underset{i=1}{\sum }}\lambda (i)+\overset{N-1}{\underset{i=1}{\sum }}\overset{N}{\underset{g=i+1}{\sum }}\lambda (i,g)\right].$$

Using Equations (48) and (49) leads to:

(64) $$\partial_{t}U_{2}{|}_{t_{0}}=\sum_{N}\int^{\text{​}}d\left(1\right)\ldots d\left(N\right){\tilde{P}}_{\left\{N\right\}}^{\left(2\right)}\;L\;\left[ln\left({\tilde{P}}_{\left\{N\right\}}^{\left(2\right)}\right)-\;\lambda \prime \right].$$

By means of boundary conditions (53), the final result is obtained:

(65) $$\partial_{t}U_{2}{|}_{t_{0}}\;=\;-\sum_{N}\int^{\text{​}}d\left(1\right)\ldots d\left(N\right)\;\;L\;{\tilde{P}}_{\left\{N\right\}}^{\left(2\right)}=0.$$

This result reminds us of the constancy of the Gibbs entropy (10), a consequence of the Liouville equation, which describes the time evolution of *P*(1,…, *N*). Similarly, Equation (65) is a consequence of BBGKY equations (58) and (59) which are derived from the Liouville equation [14].

It should be emphasized that the time derivative of *U*_2_ vanishes only at the time *t*_0_, where the system has no three-body correlation. The equation $\partial_{t}U_{2}{|}_{t_{0}}=0$ is clearly a necessary condition for *U*_2_ to always keep the same value.

The three following clues are likely to convince that the condition is also sufficient:

- The development of ternary correlations would mean that the probability densities depend on a three-variable function *λ*(1,2,3) that cannot be built from the first two Lagrange multipliers. In the absence of the three-body potential term, there is no source for any three-variable function in the system.
- From a more physical point of view, we know that the expected equilibrium Phase Space Density of a system of molecules interacting through a pairwise additive potential *Vij* is given by Equation (9). Due to its structure, this expression does not include any three-body correlation term and it is hardly conceivable that during the time evolution of the system, three-body correlations would be built by the molecular interactions and finally would be destroyed by the same interactions for asymptotic times.
- According to Equation (65), when starting from a state without three-body correlations, no ternary correlation appears after an infinitesimal time interval. This moment can then be considered as a new initial time, still without three-body correlations and, step by step, we concluded that the three-body correlations can never appear in such a system.

In the absence of formal proof, we will refer to this result as the Main Conjecture, namely: If a system is free of correlations of order greater than two at some time, its uncertainty based on the first two RDFs remains constant if its Hamiltonian involves only two-body interaction terms. In other words: No three-body correlations can be created by a Hamiltonian without three-body or many-body interaction terms.

## 8. Discussion and Conclusions

### 8.1. Summary of the Method of Defining Correlations

As the first stage of defining *s*-order correlation, the *s*-body correlation-free distribution must be defined. A system is free of *s*-body correlations whether the knowledge of the *s*-th RDF does not give more information than that included in the lower order RDFs. If so, the *s*-th RDF can be expressed in terms of the lower order RDFs. 

Accordingly, the distribution function without *s*-body correlations must maximize uncertainty and admit the (*s*−1)-body RDFs as marginal distribution functions. This is the MAXimum ENTropy Method [3], well-known as a method for taking into account some available information without introducing additional information. However, the method is applied here in a non-equilibrium context and the constraints concern marginal probability density functions rather than mean values. 

This way of defining correlations is valid in a continuous probability space as well as in a discrete probability space and there is a close analogy between the two cases. In both cases, no closed-form expression of the uncorrelated three-body distribution function in terms of the marginal (density) probabilities can be given. In the discrete case, a numerical solution exists and in statistical mechanics, the solution is given as a diagrammatic series expansion.

We should emphasize that, in order to have a uniform vocabulary for binary correlations and higher-order correlations, what we call in this article “a distribution without binary correlations” is usually called “a distribution of independent variables”.

### 8.2. The Proper Way to Measure Correlations

The correlation of *k*-order is defined as the difference between the information available from the marginal distributions of *k*-order and the information contained in the marginal distributions of (*k*−1) order. In other words, the measure of correlation of *k*-order is the difference between the uncertainties calculated from the *k*-order marginal distributions and the same expression calculated from the *k*−1 marginal distributions:

(66) $$\mathrm{Measure}\mathrm{of}k\mathrm{order}\mathrm{correlations}=\;U_{k-1}-U_{k}\geq \;0.$$

For discrete distributions, it writes:

(67) Uk−1−Uk=U[p˜i….(k−1)]−U[p˜i….(k)]=DKL(p˜i…(k)||p˜i…(k−1))≡∑i…p˜i….(k)ln(p˜i….(k)p˜i….(k−1))≥0

This measure of correlation of *k*-order appears as the average value of a logarithmic term over all possible values of the variables. The logarithmic term itself is a measure of the local correlation since it measures the gap between the full *k*-dimensional probability table [or the *k*-body RDFs] and the corresponding non-correlated expression constructed from the (*k*−1)-dimensional probability table [or the (*k*−1)-body RDFs]. This definition of correlations is proved to be fruitful [5,6].

This connected information of order *k* is identical to the "mutual information" only if *k* = 2. Due to the positivity property of the connected information of any order, this measure of correlations avoids the difficult interpretation of negative correlations as it occurs when the definition of the correlations is based on mutual information [23].

### 8.3. About the Main Conjecture

Gravitational globular clusters are the only observable systems for which one is sure that the potential is pairwise additive. Unfortunately, due to the size of the constituent units of these systems, neutron or X-ray scattering experiments are impossible and the two-body RDF, as well as the binary correlations, cannot be determined. So, it is impossible to confirm the conjecture (i.e., the constancy of *U*_2_) by this means.

From the Main conjecture, we deduce that creating the three-body correlations requires at least a three-body potential. So, the original Information-theoretic definition of correlations is transformed into a dynamical definition of correlations: the s-body correlations are those created by potentials of order-*s*.

If the main conjecture is true, it can be generalized and extended to the three-body correlations as an example: If a system of particles is described by a Hamiltonian including only two- and three-body potential terms, then uncertainty based on the first three RDFs, U_3_ is conserved. 

### 8.4. About Irreversibility

In a system of particles governed by a weak two-body potential, the global uncertainty *U* may be decomposed into a main part: the perfect gas uncertainty *U*_1_ and a smaller part related to higher order RDFs. We see immediately that this negative small part (= *U* − *U*_1_) is the opposite of the measure of correlations. Even though the two contributions have very different values, their sum U must remain constant and the increase of *U*_1_ is the opposite of the increase of *U* − *U*_1_. This statement generalizes a former result obtained at the lowest order in the density in a canonical ensemble formalism [24]. The perfect gas uncertainty *U*_1_ increases while correlations develop. As far as the potential is sufficiently weak for the correlations that it creates to be negligible, uncertainty *U*_1_ tends towards an equilibrium value which is the entropy of the system (up to a constant factor). Thus, *U*_1_ is a good indicator to study the irreversibility of weakly correlated systems as Boltzmann did it.

If the potential is not weak or if the gas is dense, *U*_1_ cannot be used as the expression of the uncertainty since *U*_1_ neglects all correlations. On the other hand, *U*_2_ takes into account the correlations and it increases when high order correlations are created. However, according to our conjecture, *U*_2_ does not increase if the intermolecular potential is pairwise additive.

Abandoning the assumption of pairwise additivity seems to be the best way out of this deadlock. Consider a dynamical system described by a Hamiltonian with a two-body potential accompanied by a weak three-body interaction term. The first two RDFs can be determined from experiment and only *U*_1_ and *U*_2_ can be determined. U_2_ shows increasing behaviour due to the creation of ternary correlations. The physical system appears irreversible. The three-body correlation uncertainty (*U* − *U*_2_) decreases while *U*_2_ increases and their sum *U* remains constant. Moreover, *k_B_U_2_* tends asymptotically to an equilibrium value which is a good approximation of entropy as far as the three-body interaction term (and all higher order potential terms) are small.

In this view, the three-body potential term does no more appear to be an improvement of the theory based on a two-body potential but rather an unavoidable necessity to explain the increase of *U*_2_. Most of the time, two-body potentials are used. Once correctly parameterized from the thermodynamic data, two-body potentials provide valuable numerical results on atoms with closed-shell structures (i.e., rare gases). However, this first-order approximation is unsuitable for other atoms and produces results that are incompatible with many experiments due to the neglect of multi-body interactions [25,26]. Moreover, unlike other transport coefficients, the first non-vanishing contribution for bulk viscosity of argon, for example, only appears when at least three-atom collisions are considered [27]. Thus, three-body interactions probably exist in all molecular systems.

### 8.5. General Conclusion

Nowadays, molecular systems with many-body potentials are more and more considered as well as the many-body correlations they produce. However, it was impossible to unravel these correlations before having a clear definition of the correlations of each order. Partitioning uncertainty (and entropy) into connected information of various orders allows describing the correlations which are created during the temporal evolution of simple thermodynamic systems as well as their reversible and irreversible characteristics.

Finally, the vision that we have about the irreversibility of a real gas system is as follows: The intermolecular potential has a two-body part and at least a small three-body part. The effects of this small three-body part are so weak that most of the time, either they are not detected or their effects are attributed to the two-body potential by having adjusted the parameters of the two-body model to have the optimal match with thermodynamic data. The relevant uncertainty for dense gases is the functional *U*_2_ which increases to the equilibrium entropy value. 

If we ever have the means to measure the three-body RDF, the uncertainty of the system would be *U_3_* and it would be constant (unless the potential involves a four-body part). In other words, the reversible aspects of the system lie in the higher-order correlations that cannot be observed.

### 8.6. Prospects

The equations of the BBGKY hierarchy could also be used in order to evaluate the asymptotical increase of *U*_2_ in systems governed by a Hamiltonian including at least a three-body potential term. We are convinced that in this context also, the parallelism with the theory of the lower order could provide useful guidelines.

In order to confirm the main conjecture of this article, three important points could be tested by means of molecular dynamics techniques:

- Showing that *U*_2_ is conserved for systems with a simple two-body potential (e.g., hard-core potential or exponential potential).
- Showing that *U*_3_ is conserved and *U*_2_ increases if an additional weak three-body potential is involved.
- We did not examine what would happen if initial correlations existed. This point should also be investigated.

## Appendix A. Uncertainty in Terms of f(1) and f(1,2) for Plasma Systems

The expression of uncertainty is available in the literature for a particular system: Laval, Pellat and Vuillemin derived it from (10) for a plasma system, using the relevant assumptions for plasmas [28].

The simplest model to study plasmas considers it as an electrically neutral medium of unbound positive ions and electrons with null overall charge. The interaction potential is of course the long-range Coulomb potential. We call *N_D_* the number of charge carriers within the Debye sphere surrounding a given charged particle. By definition, *N_D_* is sufficiently high as to shield the electrostatic influence of the particle outside of the sphere. To express the weakness of the potential, we introduce a multiplicative small parameter *ϵ*. Any link *C_ij_* is also proportional to ϵ.

Each diagram with *N* points involves an integral on its centre of mass and *N* − 1 integrals over *N* − 1 relative coordinates. An integral of a link between particles *i* and *j* over the relative coordinate *r_ij_* gives a contribution proportional to both *ϵ* and *N_D_*. As the number of interacting particles *N_D_* is high, the product *ϵ*.*N_D_* is not negligible. On the contrary, the contribution arising from a double link between the same points has a supplementary *ϵ* factor and is negligible; only ring diagrams  have to be retained. In the same way, the two-particle terms simply reduce to $-\frac{1}{4}\int^{\text{​}}d\left(1\right)d\left(2\right)f\left(1\right)f\left(2\right)\;(C_{12})²$.

The uncertainty then reduces to the expression obtained by Laval, Pellat and Vuillemin for the nonequilibrium entropy of plasmas by means of a completely different method [29]:

(A1)

## Appendix B. Expressions of the Higher-Order RDFs in Terms of f(1) and f(1,2)

The full diagrammatic expansion of ${\tilde{f}}_{\left(1,2,3\right)}^{\left(2\right)}$ (and all higher-order RDFs) was provided by Blood [19]:

(A2) $$\;{\tilde{f}}_{\left(1,2,3\right)}^{\left(2\right)}=\frac{\;f\left(1,2\right)f\left(1,3\right)f\left(23\right)}{f\left(1\right)f\left(2\right)f\left(3\right)}\;\times \;exp\;\left(\;B_{3}\;\right)$$

where *B*_3_ is defined as the sum of all the basic diagrams with three white circles labelled 1, 2 and 3. (A basic diagram is a connected diagram in which there is at least one path to reach a white circle from any circles even if a black circle is removed).

The first few terms of the expansion of ${\tilde{f}}_{\left(1,2,3\right)}^{\left(2\right)}$ can be obtained by variating *U*_2_ with respect to RDFs and equating the result to zero:

(A3)

## Appendix C. Expressions of λ(1) and λ(1,2) in terms of f(1) and f(1,2)

Lagrangian (26) may also be presented in terms of RDFs:

(A4) $$L\;=\;U-\int^{\text{​}}d\left(1\right)\;\lambda \left(1\right)\left[f_{\left(1\right)}^{given}-f\left(1\right)\right]\;-\;\int^{\text{​}}d\left(1\right)d\left(2\right)\frac{\lambda \left(1,2\right)}{2}\left[f_{\left(1,2\right)}^{given}-f\left(1,2\right)\right],$$

and it can be maximized with respect to *f*(1) and *f(1,2)*. This leads to two formal equations:

(A5) λ(1) = − δUδf(1);

(A6) λ(1,2) =−2[δUδf(1,2)].

Let us first take the functional derivative of the first two integrals of Equation (37)

(A7) $$\frac{\delta \left(two\;first\;integrals\;of\;U\right)}{\delta f\left(1\right)}\;=\;-ln\left[h^{3}f\left(1\right)\right]-\frac{1}{2}\int^{\text{​}}d\left(2\right)f\left(2\right)\left[-\frac{2f\left(1,2\right)}{f\left(1)f(2\right)}+2\right]$$

The functional derivative of the first diagram is more involved:

(A8) $$\begin{array}{cc} \frac{\delta \Delta }{\delta f\left(1\right)}= & \frac{\delta }{\delta f\left(1\right)}\left\{\frac{1}{6}\int^{\text{​}}d\left(1\right)d\left(2\right)d\left(3\right)f\left(1\right)f\left(2\right)f\left(3\right)\left[C_{12}C_{13}C_{23}\right]\right\}\\ & =\frac{1}{6}\int^{\text{​}}d\left(2\right)d\left(3\right)\left[3f\left(2\right)f\left(3\right)C_{12}C_{13}C_{23}\;+3f\left(1\right)f\left(2\right)f\left(3\right)C_{13}C_{23}\left(\frac{\delta C_{12}}{\delta f\left(1\right)}\right)\right]\\ & =\frac{3}{6}\int^{\text{​}}d\left(2\right)d\left(3\right)\;f\left(2\right)f\left(3\right)C_{13}C_{23}\left[C_{12}+f\left(1\right)\left(-\frac{2f\left(1,2\right)}{f²\left(1)f(2\right)}\right)\right]\\ & =\frac{1}{2}\int^{\text{​}}d\left(2\right)d\left(3\right)\;f\left(2\right)f\left(3\right)C_{13}C_{23\;\;}\left[-C_{12}-2\right] \end{array}$$

Equations (A5),(A7) and (A8) give:

(A9) $$\lambda (1)=ln\left[h^{3}f\left(1\right)\right]-\int^{\text{​}}d\left(2\right)f\left(2\right)\;C_{12}+\frac{1}{2}\int^{\text{​}}d\left(2\right)d\left(3\right)\;f\left(2\right)f\left(3\right)\left[C_{12}C_{13}C_{23}+2C_{13}C_{23}\right]+\ldots \;.$$

To derive functionally Equation (37) with respect to *f*(1,2), we first notice that we may derive the second and third lines of Equation (37) with respect to *C*_12_ due to a consequence of the chain rule:

(A10) δ…δf(1,2)=1f(1)f(2)[ δ…δC12]

(A11) λ(1,2)=−2δUδf(1,2)=−2[−12lnf(1,2)f(1)f(2)+12∫​d(3) f(3)C13C23+124∫​d(3)d(4)f(3)f(4)[−12C14C23C34+6 C13C14C23C24C34]]=lnf(1,2)f(1)f(2)−∫​d(3) f(3)C13C23 +12∫​d(3)d(4)f(3)f(4) C34[ 2C14C23− C13C14C23C24]+… .

The full diagrammatic expression of *λ(1,2)* is:

(A12) $$\lambda (1,2)\;=\;ln\;\frac{f\left(1,2\right)}{f\left(1\right)f\left(2\right)}-N_{2}-B_{2},$$

where $N_{2}$ and $B_{2}$ (called nodal and basic diagrams, respectively) are obtained from diagrams  and  by replacing 2 black circles with white circles, labelling them “1” and “2” and erasing their link.

Also this result is available for equilibrium conditions [19,29].

## Appendix D. Basic and Generalized Kirkwood Superposition Approximation

When Lagrangian (A4) is differentiated with respect to the RDFs, the first terms of the series expansion of the two Lagrange multipliers *λ*(*i*) and *λ*(*i*,*j*) are obtained step by step.

To perform the functional derivatives of *U* with respect to the RDFs, the expression of *U* in terms of the RDFs must be used [9,15]:

(A13) $$\begin{array}{cc} U= & -\int^{\text{​}}d\left(1\right)\left[f\left(1\right)\ln h^{3}f\left(1\right)–f\left(1\right)\right]\;-\;\frac{1}{2}\int^{\text{​}}d\left(1\right)d\left(2\right)\left[f\left(1,2\right)\ln\frac{f\left(1,2\right)}{f\left(1\right)f\left(2\right)}-f\left(1,2\right)+f\left(1\right)f\left(2\right)\right]\\ & -\frac{1}{3!}\int^{\text{​}}d\left(1\right)d\left(2\right)d\left(3\right)f\left(1,2,3\right)\left(\ln\frac{f\left(1,2,3\right)\;f\left(1\right)f\left(2\right)f\left(3\right)}{f\left(1,2\right)f\left(2,3\right)f\left(3,1\right)}\right)\\ & +\frac{1}{3!}\int^{\text{​}}d\left(1\right)d\left(2\right)d\left(3\right)\left[\;f\left(1,2,3\right)-3f\left(1,2\right)f\left(2,3\right)/f\left(2\right)+3f\left(1,2\right)f\left(3\right)\;-f\left(1\right)f\left(2\right)f\left(3\right)\right]\\ & -\frac{1}{4!}\int^{\text{​}}d\left(1\right)d\left(2\right)d\left(3\right)d\left(4\right)f\left(1,2,3,4\right)\left(\ln\frac{\;\;f\left(1,2,3,4\right)\;.\;\;f\left(1,2\right)f\left(1,3\right)f\left(1,4\right)f\left(2,3\right)\;f\left(2,4\right)\;f\left(3,4\right)}{f\left(1,2,3\right)\;f\left(1,2,4\right)\;f\left(1,3,4\right)f\left(2,3,4\right)\;.\;\;f\left(1\right)f\left(2\right)f\left(3\right)f\left(4\right)}\right)\\ & +\frac{1}{4!}\int^{\text{​}}\begin{array}{c} d\left(1\right)d\left(2\right)d\left(3\right)d\left(4\right)\left[\;\begin{array}{c} f\left(1,2,3,4\right)-6f\left(1,2,3\right)f\left(1,2,4\right)/f\left(1,2\right)+12f\left(1,2\right)f\left(2,3,4\right)/f\left(2\right)\\ -4f\left(1,2,3\right)f\left(4\right)-4f\left(1,2\right)f\left(1,3\right)f\left(1,4\right)/f²\left(1\right)-3f\left(1,2\right)f\left(3,4\right)\\ \;\;+6f\left(1,2\right)f\left(3\right)f\left(4\right)-2f\left(1\right)f\left(2\right)f\left(3\right)f\left(4\right) \end{array}\right]-\ldots \end{array} \end{array}$$

The functional derivatives of the Lagrangian (A4) with respect to the three- and the four-body RDF must be cancelled out:

(A14) $$0=-\frac{1}{3!}\ln\frac{f\left(1,2,3\right)\;f\left(1\right)f\left(2\right)f\left(3\right)}{f\left(1,2\right)f\left(2,3\right)f\left(3,1\right)}\;+\frac{1}{4!}\int^{\text{​}}d\left(4\right)\;4\left[\frac{f\left(1,2,3,4\right)}{f\left(1,2,3\right)}-\frac{f\left(1,2,4\right)}{f\left(1,2\right)}-\frac{f\left(1,3,4\right)}{f\left(1,3\right)}-\frac{f\left(2,3,4\right)}{f\left(2,3\right)}+\frac{f\left(2,4\right)}{f\left(2\right)}+\frac{f\left(1,4\right)}{f\left(1\right)}+\frac{f\left(3,4\right)}{f\left(3\right)}-f\left(4\right)\right]+\ldots \;$$

and

(A15) $$0\;=ln\frac{\;f\left(1,2,3,4\right)\;.\;f\left(1,2\right)f\left(1,3\right)f\left(1,4\right)f\left(2,3\right)\;f\left(2,4\right)\;f\left(3,4\right)}{f\left(1,2,3\right)\;f\left(1,2,4\right)\;f\left(1,3,4\right)f\left(2,3,4\right)\;.\;f\left(1\right)f\left(2\right)f\left(3\right)f\left(4\right)}+\;\ldots \;.$$

The Fisher-Kopeliovich closure results directly from Equation (A15).

Due to this closure for the quadruplet function, the second and the third BBGKY hierarchy becomes a pair of nonlinear integro-differential coupled equations for *f*(1,2) and *f*(1,2,3). In some applications, the quadruplet function itself is required [30].

The Kirkwood superposition approximation (KSA) [20] is the solution of Equation (A14) at lowest order. It was extensively used to make the second BBGKY hierarchy equation solvable: After substitution, the pair correlation function is the only function to be determined in this equation.

If the integral in Equation (A14) is evaluated by means of the Fisher-Kopeliovich closure and KSA, the first correction to the KSA is achieved. The KSA thus appears as the first order in the density of the expression of *f*(1,2,3) that contains no other information than the one contained in *f*(1) and *f*(1,2).

Appendix E shows that a generalized superposition approximation (for *n* ≥ 4) can also be obtained by maximizing the uncertainty and it appears again to be the leading term of the expression of the *n*-th RDF when it is expressed exclusively in terms of the lower order RDFs.

If the number of particles is considered fixed (canonical ensemble), additional normalization factors appear. They are similar to those mentioned in Equation (19). These normalization factors disappear and the KSA is obtained only when the thermodynamic limit (*N* → ∞, *V* → ∞, *N / V* = Constant) is applied. This explains the role played by the thermodynamic limit in the derivation of KSA published by Singer [10], which is also based on a maximum principle under the constraints expressed by the definitions of the first two RDFs. Such a limit is not required in the grand canonical formalism.

Moreover, uncertainty is the unique expression to be maximized in the grand canonical formalism whereas, in Singer’s derivation [10], the maximized expression depends on the desired target, namely, either “the triplets entropy” for the KSA or “the quadruplets entropy” for the Fisher-Kopeliovich closure.

## Appendix E. Generalized Kirkwood Superposition Approximation

Uncertainty may be written as a series expansion where each term involves one more particle than the previous one [8,9,16]:

(A16) $$U\;=-3\langle N\rangle ln\;h\;+\underset{s\geq 0}{\sum }U^{\left(s\right)},$$

with

(A17) $$U^{\left(s\right)}\;=-\int^{\text{​}}\frac{d\left(1\right)\ldots d\left(s\right)}{s!}f\left(1,\ldots ,s\right)\;\;ln\underset{1\leq N\leq s}{\prod }\underset{\;\;\;\;0<i_{1}<i_{2}<<i_{N}\leq s}{\prod }f{\left(i_{1},i_{2},\ldots ,i_{N}\right)}^{{\left(-1\right)}^{s-N}}\;+\;\int^{\text{​}}\frac{d\left(1\right)\ldots d\left(s\right)}{s!}\left\{f\left(1,\ldots ,s\right)\;+\;F\left[f\left(1\right),f\left(2\right),\ldots ,f\left(s-1\right)\right]\right\},$$

where *F*[*f*(1), *f*(2),..., *f*(*s* − 1)] represents a functional of all RDFs that involve less than s particles.

In order to express *f*(1,…, *s*) in terms of the lower order RDFs, we have to take the functional derivative of *U* and its components $U^{\left(s\right)}$ with respect to *f*(1,…, *s*):

(A18) $$\frac{\delta U^{\left(s\right)}}{\delta f\left(1,\ldots ,s\right)}\;=\;\frac{1}{s!}\left[-ln\underset{1\leq N\leq s}{\prod }\underset{\;0<i_{1}<i_{2}<<i_{N}\leq s}{\prod }f{\left(i_{1},i_{2},\ldots ,i_{N}\right)}^{{\left(-1\right)}^{s-N}}-\frac{f\left(1,\ldots ,s\right)}{f\left(1,\ldots ,s\right)}+1\right]$$

The functional derivatives of all components *U^(z)^* with respect to *f*(1,…, *s*) involve more than *s* particles if *z* > *s*. As a density factor is associated to each particle, these terms may be neglected if we retain only the lowest order in the density. Requiring that the functional derivative of *U*^(*s*)^ with respect to *f*(1,…, *s*) vanishes, leads to the generalized superposition approximation [20]:

(A19) $$\underset{1\leq N\leq s}{\prod }\underset{\;0<i_{1}<i_{2}<<i_{N}\leq s}{\prod }f{\left(i_{1},i_{2},\ldots ,i_{N}\right)}^{{\left(-1\right)}^{s-N}}=1.$$

This equation can be solved with respect to *f*(1,…, *s*):

(A20) $$f\left(1,\ldots ,s\right)=\underset{1\leq N\leq \;s-1}{\prod }\underset{\;0<i_{1}<i_{2}<<i_{N}\leq s}{\prod }f{\left(i_{1},i_{2},\ldots ,i_{N}\right)}^{{\left(-1\right)}^{s-N-1}},$$

or in a more synthetic notation:

(A21) $$f\left(1,\ldots ,s\right)=\underset{\left\{N\right\}\;\subseteq \;\left\{s-1\right\}}{\prod }f{\left(1,\ldots ,N\right)}^{{\left(-1\right)}^{s-N-1}}$$

where the product over {N}⊆{s−1} contains all different subsets of coordinates {*N*} in {*s* − 1}.

So, the *n*-th superposition approximation appears to be the leading term of the expression of the *n*-th RDF when it is expressed exclusively in terms of the lower order RDFs as it may be constructed from information theory.
